# Policy stakeholder perspectives on barriers and facilitators to launching a community-wide mass drug administration program for soil-transmitted helminths

**DOI:** 10.1186/s41256-022-00281-z

**Published:** 2022-12-02

**Authors:** Amy Roll, Malvika Saxena, Elizabeth Orlan, Angelin Titus, Sanjay Kamlakar Juvekar, Marie-Claire Gwayi-Chore, Euripide Avokpaho, Félicien Chabi, Comlanvi Innocent Togbevi, Abiguel Belou Elijan, Providence Nindi, Judd L. Walson, Sitara S. R. Ajjampur, Moudachirou Ibikounlé, Khumbo Kalua, Kumudha Aruldas, Arianna Rubin Means

**Affiliations:** 1 Department of Global Health, University of Washington, Seattle, WA USA; 2 The Wellcome Trust Research Laboratory, Division of Gastrointestinal Sciences, Christian Medical College, Vellore, India; 3 Vadu Rural Health Program, KEM Hospital Research Centre, Pune, India; 4 Institut de Recherche Clinique du Bénin, Abomey-Calavi, Bénin; 5 Blantyre Institute for Community Outreach, Blantyre, Malawi; 6 Departments of Medicine, Pediatrics, and Epidemiology, University of Washington, Seattle, WA USA; 7 Centre de Recherche pour la lutte contre les Maladies Infectieuses Tropicales (CReMIT/TIDRC), Université d’Abomey-Calavi, Godomey, Bénin

**Keywords:** Soil-transmitted helminths, Neglected tropical diseases, Health policy, Facilitators, Barriers, Mass drug administration, Implementation science, Policy guidelines

## Abstract

**Background:**

Recent evidence suggests that soil-transmitted helminth (STH) transmission interruption may be feasible through community-wide mass drug administration (cMDA) that deworms community members of all ages. A change from school-based deworming to cMDA will require reconfiguring of STH programs in endemic countries. We conducted formative qualitative research in Benin, India, and Malawi to identify barriers and facilitators to successfully launching a cMDA program from the policy-stakeholder perspective.

**Methods:**

We conducted 40 key informant interviews with policy stakeholders identified as critical change agents at national, state/district, and sub-district levels. Participants included World Health Organization country office staff, implementing partners, and national and sub-national government officials. We used the Consolidated Framework for Implementation Research to guide data collection, coding, and analysis. Heat maps were used to organize coded data and differentiate perceived facilitators and barriers to launching cMDA by stakeholder.

**Results:**

Key facilitators to launching a cMDA program included availability of high-quality, tailored sensitization materials, and human and material resources that could be leveraged from previous MDA campaigns. Key barriers included the potential to overburden existing health workers, uncertainty of external funding to sustain a cMDA program, and concerns about weak intragovernmental coordination to implement cMDA. Cross-cutting themes included the need for rigorous trial evidence on STH transmission interruption to gain confidence in cMDA, and implementation evidence to effectively operationalize cMDA. Importantly, if policy stakeholders anticipate a cMDA program cannot be sustained due to cost and human resource barriers in the long term they may be less likely to support the launch of a program in the short term.

**Conclusions:**

Overall, policy stakeholders were optimistic about implementing cMDA primarily because they believe that the tools necessary to successfully implement cMDA are already available. Policy stakeholders in this study were cautiously optimistic about launching cMDA to achieve STH transmission interruption and believe that it is feasible to implement. However, launching cMDA as an alternative policy to school-based deworming will require addressing key resource and evidence barriers.

*Trial registration* This study was registered in the U.S. National Library of Medicine Clinical Trials registry (NCT03014167).

**Supplementary Information:**

The online version contains supplementary material available at 10.1186/s41256-022-00281-z.

## Background

Soil-transmitted helminths (STH) are a group of intestinal worms that affect an estimated 1.5 billion people annually who primarily reside in low- and middle-income countries (LMICs) [1]. STH is one of the most common infections affecting impoverished communities, particularly those with inadequate access to safe water and sanitation. Moderate to heavy intensity infection with STH is associated with anemia, growth stunting, and impaired physical and cognitive development [1–3]. Current World Health Organization (WHO) guidelines for STH prioritize morbidity control amongst populations at high risk for developing STH-associated morbidities, including pre-school and school-age children, women of reproductive age, pregnant women, and adults in high-risk occupations [2]. The current standard of care for STH is school-based deworming through mass drug administration (MDA) of anthelmintic drugs such as albendazole and mebendazole. However, the school-based MDA model leaves several populations at risk of being untreated, including school-aged children who are not enrolled in school, their parents, and other community members at risk of STH infection. These populations serve as infection reservoirs in the community putting children and other susceptible groups at ongoing risk of reinfection. If reservoirs of STH infection persist in the community, school-based MDA might need to be sustained indefinitely [4–6].

Early evidence suggests that it might be possible to interrupt transmission of STH early using community-wide MDA (cMDA), as opposed to school-based MDA [7–9]. Many neglected tropical disease (NTD) programs, such as lymphatic filariasis (LF) programs, have successfully used cMDA to interrupt disease transmission [10, 11]. In a cMDA program for STH, community members of all ages are dewormed, typically via door-to-door or fixed-point delivery through a volunteer workforce of community drug distributors (CDDs) [12]. However, a change in global STH guidelines from morbidity control amongst children to transmission interruption at a community level will require reconfiguring STH programs at a national level in STH-endemic countries. Transitioning platforms from targeting children through school-based MDA to platforms that reach the entire community would potentially require adapting school-based MDA sensitization resources and activities to reach all community members; transferring administration of STH MDA programs from Ministries of Education to Ministries of Health, or establishing a coordination mechanism between the two agencies; training volunteer CDDs to deliver cMDA; and strengthening supply chains for the increased quantity of deworming drugs needed for cMDA [13].

For programs to successfully make this transition, it is important to understand factors that influence the launch of cMDA for STH transmission interruption [6]. Previous studies have found that early involvement of policy stakeholders has a positive effect on roll out of new community-level intervention policies in LMICs [14]. Further, understanding the buy-in and support (or lack thereof) of policy stakeholders can help to proactively address implementation challenges, such as changes in infrastructure needed to implement a new intervention [14–16]. We conducted formative qualitative research with key STH policy stakeholders in three countries (Benin, India, and Malawi), prior to the rollout of a new cMDA trial for STH transmission interruption. These stakeholders included national-level government officials, implementing partners, WHO, and state/district level Ministry of Health (MOH) officers.

The purpose of this study is to identify policy-level determinants (facilitators and barriers) of launching cMDA across three heterogeneous contexts in Benin, India, and Malawi. This evidence is necessary for identifying best practices for rollout of cMDA as part of larger STH transmission interruption programs, or other newly launched community-based public health campaigns.

## Methods

This qualitative study is embedded within the DeWorm3 Project, a hybrid type 1 implementation-effectiveness trial evaluating the feasibility of interrupting STH transmission using cMDA [12, 17–19]. The DeWorm3 trial aims to evaluate the feasibility of interrupting transmission of STH using biannual cMDA targeting community members of all ages [12]. Additionally, DeWorm3 implementation science (IS) research aims to evaluate the epidemiological, intervention characteristics, systems factors, and social factors influencing cMDA to develop and test a model that is sustainable and scalable [17]. This formative qualitative study included data collection at project baseline, prior to the launch of the DeWorm3 trial. This study was designed to gain insight into policy-level factors influencing the initial rollout of cMDA.

### Study setting

DeWorm3 trial sites include Benin, India, and Malawi Site selection is described in more detail in the DeWorm3 trial protocol [12]. In Benin the current standard-of-care is school-based MDA that targets school-aged children (SAC) between 5 and 14 years of age. In India, the standard-of-care includes school-based MDA and National Deworming Days that target preschool-aged children (PSAC) and SAC 1–19 years of age. In Malawi, the standard-of-care includes school-based MDA and Child Health Days that target PSAC and SAC ages 1–14 years old. More information about baseline prevalence and other site characteristics can be found in detail elsewhere [18].

### Study population and sampling

Key policy stakeholders—including MOH, Ministry of Education, WHO and other implementing partners such as non-governmental organizations (NGOs)—play an important role in shaping, adopting, and scaling up new NTD programs and policies. Stakeholder mapping workshops were conducted in each country to identify individuals who were considered critical STH change agents in each country at the national, state/district, and sub-district levels. Stakeholder maps included 52 individuals in Benin, 137 individuals in India, and 54 individuals in Malawi. Purposive quota sampling was used to select interviewees from the stakeholder maps across stakeholder “levels”, including WHO country offices, implementing partners, and national and sub-national government personnel in each country [20]. We aimed to sample two to three individuals at the Implementing Partner and National level and five to ten individuals at the sub-national level.

### Data collection

In 2018, we conducted individual interviews with STH policy stakeholders in Benin, India, and Malawi, before the rollout of cMDA in conjunction with the DeWorm3 Project. Data collection and analysis were informed by the Consolidated Framework for Implementation Research (CFIR), a meta-theoretical framework of 38 constructs that provides a typology for characterizing potential determinants (both barriers and facilitators) to implementation from the perspective of individuals involved in implementation [21]. We identified a priori 24 CFIR constructs to address in this study, which were used to shape three semi-structured interview guides. The interview guides contained a mix of respondent and informant style questions, tailored to stakeholder group at national/state level, district level, and sub-district levels. Site-adapted question guides were translated into the national and/or local languages, including French (Benin) and Tamil (India). Study staff fluent in the local languages were trained to conduct interview and they conducted interviews in locations convenient to respondents. Prior to starting the interview, all participants provided written informed consent or indicated their consent using a thumb print in the presence of a witness. Following consenting procedures, interviews were audio-recorded.

### Analysis

Data were transcribed in the local languages, where applicable, and translated into English. Transcriptions and translations underwent quality assurance reviews to ensure accuracy and any discrepancies were referred to the original transcriber or translator for revision. Participant names were removed from transcripts but the stakeholder level (e.g., national-level) was included. Atlas.ti 8 qualitative software was used to store and organize the transcripts. A group of five coders based in Seattle, United States; Vellore, India; and Cotonou, Benin engaged in coding the data. All coders were trained using a standardized analysis plan. Data were analyzed deductively using an a priori codebook drawing from 24 CFIR constructs across all five domains, three CFIR constructs that were duplicated to specify if they were relevant to STH or other NTDs (LF), and five additional codes (non-CFIR) that we hypothesized influenced the implementation of cMDA (Additional file 1). Codebook definitions and inclusion and exclusion criteria were updated iteratively by coders during the coding process. Most transcripts were assigned to two coders for double coding. A subset (all transcripts from Malawi and a subset of seven transcripts from India) were assigned only to a single coder due to availability. Coders met weekly to discuss variability in coding and to resolve differences. An additional third coder reviewed discrepancies in codes and served as a tiebreaker when necessary.

Case memos were developed for each stakeholder group by site and were further summarized within an Excel-based heat map. Heat maps were used to organize coded data by CFIR construct and differentiate perceived facilitators and barriers by stakeholder. Where individual responses indicate that a construct makes the introduction of cMDA easier (i.e., a facilitator), their response was designated as a strong (dark blue) or moderate (light blue) facilitator. Where the individual indicated that a construct makes the introduction of cMDA difficult (i.e., a barrier), their response was designated as a strong (dark orange) or moderate (light orange) barrier. If the individual indicated that a construct could be both a facilitator and a barrier or was neutral (no evidence of positive or negative influence), it was designated as mixed (grey). Heat maps were visually assessed for patterns across stakeholders, stakeholder levels, constructs, and sites. Facilitators and barriers were identified by noting patterns that were cross-cutting across individuals, constructs, and/or countries and via thematic organization and analysis of coded data.

## Results

A total of 40 respondents participated in this study (Table 1). These STH policy stakeholders identified several potential facilitators and barriers influencing the effective launch of cMDA for STH. Key facilitators include ensuring there are tailored community sensitization protocols in place and that existing campaign infrastructure can be leveraged (as opposed to creating new systems). Barriers included concerns about health workers’ readiness and capacity to conduct the intervention, competing funding priorities, and concerns about existing intragovernmental partnerships. An additional cross-cutting theme was the importance of rigorous clinical and implementation evidence to inform potential transitions in the STH standard of care.

**Table 1:** Stakeholders interviewed by country and level (N = 40)

Stakeholder level	Benin	India	Malawi
WHO/Implementing partner	3	3	2
National government	2	2	4
Subnational 1st level (state/district/			
department)	2	3	8
Subnational 2nd level (sub-district/block)	4	7	0
Total	11	15	14

### Key facilitators

#### Effective, tailored community sensitization is a key component of successfully launching cMDA

**Fig. 1:**
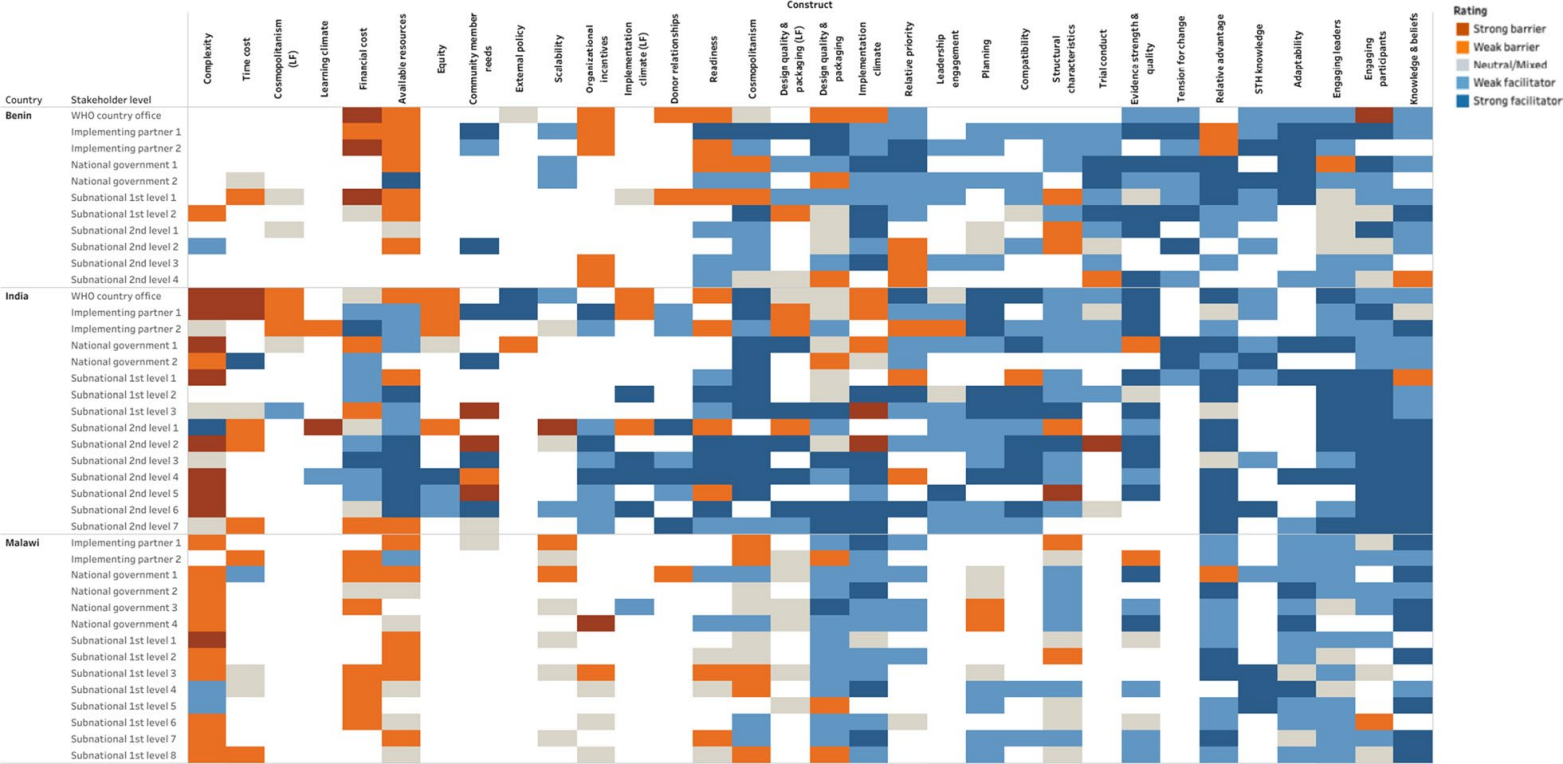
Heatmaps of policy stakeholders

Policy stakeholders indicated that the effective launch of cMDA with high coverage relies on community sensitization that dispels rumors, addresses myths, and mobilizes community members through education, training, and other similar activities. Four CFIR constructs, *engaging participants, engaging leaders, design quality and packaging,* and *intervention complexity* influenced this theme by highlighting opportunities and best practices for strong sensitization campaigns to support rollout of cMDA (Fig. 1).

Policy stakeholders across all three countries drew from their experience leading other MDA campaigns, and emphasized that participants need to be engaged through outreach activities (social marketing) before and during cMDA to overcome myths related to MDA (CFIR construct of *engaging participants*, Fig. 1). During prior campaigns, stakeholders noted that news of adverse or side effects that were unrelated to the drugs had dramatic effects on subsequent community coverage. Similarly, stakeholders were concerned that expanding MDA to adults will create new sensitization challenges for STH programs that are accustomed to compliant pediatric populations. A stakeholder from Benin mentioned that if cMDA sensitization was not tailored to address adult concerns, drug distributors “[may] be chased away” (Benin #9 (District)).

Based on their experiences with other cMDA programs for LF, stakeholders in India and Benin expressed concern that adults may reject cMDA for STH if they perceive themselves to be low risk for infection. Thus, stakeholders believed previous MDA awareness materials and social mobilization tools could be leveraged from other campaigns to overcome this challenge.

Convincing the whole community, why the whole strategy is being changed… we might have to prepare some communication messages, some advocacies so we will use all the strategies which we generally use: advocacy with the teachers, the leaders. Explain [to communities] the benefit, what is the power of this intervention, what is the gain that you are going to get. Instead of deworming for 20 years you might have to deworm only about 2 years to stop [STH transmission].—India #2 (National)

Policy stakeholders in India and Malawi believed tailored sensitization to specific community sub-groups would make cMDA more complex to deliver (CFIR construct of *intervention complexity*, Fig. 1). While complexity was largely viewed as a challenge, it was viewed as a surmountable challenge necessary to implement effective sensitization activities. In India, national and sub-district level stakeholders suggested sensitization techniques should vary by geography, education, and socioeconomic status. Stakeholders in Malawi believed cMDA sensitization would need to be adapted for different cultures and religious beliefs and should involve religious leaders in sensitization efforts or consider distribution at religious institutions.

We need to sensitize massively like going to the churches... and Mosques. As you know [in District] most of the people are Muslims, so we need to target those areas. We also need to make sure that the local leaders know this issue and we need to tell them that wherever people have gathered…it is also their responsibility to let the people know what government intends to do in their area.—Malawi #8 (District)

Stakeholders in all sites warned that each community has unique myths and rumors; effective sensitization is sensitization that can be tailored to local contexts. Policy stakeholders felt most comfortable with launching cMDA if adaptable sensitization strategies were already in place.

#### Opportunities to leverage existing health campaign infrastructure is important for policy stakeholders to support cMDA launch

Policy stakeholders believed cMDA should be launched using existing MOH resources, including human and material resources, if it is aligned with current health programs and goals. Three CFIR constructs, *structural characteristics*, *tension for change*, and *adaptability*, contributed to the development of this theme (Fig. 1).

Policy stakeholders across three countries believed existing school based and LF MDA resources could be used to support the launch of cMDA (CFIR construct of *structural characteristics*, Fig. 1). This includes training CDDs from prior MDA campaigns for other NTDs and leveraging existing infrastructure such as office space and drug storage facilities. Policy stakeholders in India focused on leveraging existing sensitization infrastructure; because albendazole is also included in LF MDA, community members may have higher acceptability of the intervention if this messaging is included in existing sensitization channels. Stakeholders in Malawi favored using existing structures to mitigate funding challenges that come with starting a new program.

The best adaptation is to utilize the existing structures because we cannot create a new structure... I am saying this because of sustainability issues because when you are doing these activities when we are starting, we may have funding but after these activities, when we want to take it in a routine way, funding might become a challenge. So, if you create a new structure, after the funding, automatically those structures will need more funding but if you use existing structures, they will use routine approach where normally they use the way they execute their duties.—Malawi #11 (National)

Policy stakeholders noted that one reason why cMDA for STH is a priority for the health system is that it is a challenge that can be addressed with tools at hand, without the need to develop new technologies or innovations.

The fact that people choose interventions is not because this is a priority, this is not a priority. It’s rather because we have the means to solve this. There are actually many things that are priorities, now if the cost of the intervention, the resources available to do this... and it does good to the population.—Benin #1 (WHO)

In Benin and India, several policy stakeholders believed it was important to pursue STH transmission interruption and launch cMDA for STH as a part of existing maternal and child health agendas (CFIR construct *tension for change)*. In Benin, several stakeholders believed children were still at risk of STH morbidities under the current standard of care, and thus STH programs need to be community wide (Fig. 1). In India, stakeholders believed cMDA for STH should be prioritized because it might effectively reduce anemia amongst women of reproductive age.

Maternal and child health is the most important program. All programs come under this program. When we look at this, one of the challenges of a pregnant woman is anemia. This anemia cannot be prevented only in pregnancy. All of a sudden after a woman gets pregnant, if anemia has to be prevented, it will not be effective… One of the effects of worms is anemia. If this is corrected in the growing age, that girl after marriage, can be healthy when she gets pregnant. Therefore, maternal mortality or child mortality can definitely be reduced because of this.—India #7 (District)

While policy stakeholders across sites highlighted the benefit of leveraging the local health workforce from previous interventions, there were still persistent concerns about having sufficient staff and training resources to manage an additional door-to-door cMDA campaign for STH.

### Key barriers

#### Policy stakeholders are concerned about health worker workload and human resources

Policy stakeholders in all sites expressed concerns about staffing availability, the potential to overburden workers, and the need for improved structured supervision should cMDA for STH become national policy. Several CFIR constructs contributed to the development of this theme, including *available resources, organizational incentives,* and *implementation climate.* Stakeholders' concerns about health worker workload were captured by the constructs: *available resources, relative advantage,* and *intervention complexity* wherein they compared the lower human resource needs and uncomplicated delivery of school-based MDA to that of cMDA. Concerns arose around immediate gaps in supervision needed for cMDA, which may reflect lower levels of readiness to launch a new cMDA program, reflected by the *readiness* construct (Fig. 1).

Policy stakeholders compared the human resource requirements of school-based MDA to cMDA, highlighting school-based deworming leverages teachers to distribute drugs, whereas in some settings CDDs would need to be recruited and trained to deliver cMDA. National-level stakeholders in Benin and Malawi did not believe there would be enough CDDs readily available to deliver cMDA for STH with high-coverage (CFIR construct *available resources,* Fig. 1). Stakeholders in all sites expressed concerns that CDDs are often overworked and underpaid, and that increasing their existing workload might result in poorly delivered cMDA programs.

The challenges could be shortage of staff because these people are doing other routine programmes and the staffing level is low. So, if you are trying to make it a routine you should also think of overloading the same staff who are understaffed. It could be better, but I think issues to do concerning staff should also be emphasized otherwise you may have challenges.—Malawi #3 (District)

While school-based MDA programs do not guarantee financial incentives to teachers to deliver deworming, policymakers in Benin and Malawi believed launching cMDA would require additional monetary incentives for CDDs or other health workers involved (CFIR construct *organizational incentives,* Fig. 1). Further, stakeholders across all levels noted that CDD motivation is often tied to their sense of value, based on the incentives received. In Benin, stakeholders were concerned that insufficient incentives and focus on performance-based targets would act as a barrier to launching cMDA with high coverage.

They say if we do not give them money, they will not distribute… some will continue to claim ‘Ah! How much is it per child?’ It’s a little absurd but we had such cases. It’s real facts, where the teacher refuses to give a free tablet to his students.—Benin #2 (Implementing partner)

Policy stakeholders in India and Malawi noted that without greatly expanding the size of the CDD workforce prior to launch, CDDs may not have sufficient time to deliver cMDA for STH. Due to financial resource restrictions, stakeholders presented scenarios in which they must choose between increasing the number of health workers participating in implementation or allocating more time to conduct cMDA.

Further, stakeholders in India and Malawi believed cMDA would present complex challenges to the CDD workforce that may make it difficult to launch the program with high coverage (CFIR construct *intervention complexity,* Fig. 1). These challenges include delivering in difficult terrain, multiple visits to reach all community members, and inadequate time (days) to administer cMDA.

Now you are increasing their task five-fold, so I don’t know how happy they are going to be…you are adding to the work and if you have to deworm everyone…it will be resource intensive, and you will find it difficult to have coverage this large, on a regular basis over a period of several years, it won’t be a simple task as compared to a school based [MDA]. In the home-based activity, you need to have a big team of people going from house to house, whereas in the school-based program you have the advantage of having teachers. You can very easily involve the teachers and your work will be reduced substantially if you have the cooperation of the education department and the teachers.—India #3 (WHO)

Policy stakeholders in Benin and Malawi were concerned that launching cMDA could overburden the existing health workforce and strain resources (CFIR construct *available resources,* Fig. 1). One policy stakeholder in Benin explained how the lack of adequate staffing may lead to burnout of CDDs and supervising health workers:

What can be problematic is that, when a single individual is used for many things, they can no longer be productive. They are exhausted and when a new intervention comes again, they cannot refuse but will do as they can. So, that's why I was speaking earlier about insufficient human resources to do the work. Because there are many things on the ground, when you go to a District health center, there may be a nurse, a midwife with caregivers…It is to such an extent that sometimes when you need them to do something, you will find them already doing something else. It is not that they are there doing nothing, no. They are busy with something. Okay, but that does not mean they are not favorable to the cause and don't understand the merits. They agree to do it, but they are limited by their temporary availability.—Benin #6 (Sub-National)

In India, most National and some sub-national policy stakeholders were concerned that the health workforce would not immediately accept launching a new cMDA program if it increased their workload (CFIR construct *implementation climate*, Fig. 1).

Naturally the workload is high for community-wide MDA, so there will not be immediate acceptance, we should give a motivation training, and should motivate on how beneficial it is, there will not be immediate acceptance, mainly because of the workload, moreover because there is less compliance at the community level, there won’t be immediate acceptance.—India #8 (Sub-National)

Policy stakeholders were similarly concerned that supervisors may not be able to provide adequate supervision during implementation, should cMDA for STH become standard of care. Supervision was of greater concern to higher level (national/WHO) stakeholders compared to district stakeholders who are typically responsible for local supervision (CFIR construct *readiness*, Fig. 1).

Very often when health workers are being trained, all I see is that they are gathered somewhere for an hour, cards are distributed without explaining anything and then they leave. But no one monitors what they do on the ground…Nobody supervises health workers; everyone thinks they are a boss at home, distributors are allowed to go to the field. Everyone does what they want. So, you have to have supervision, you need to follow the actors on the field and now be willing to manage any complications arising.—Benin # 1 (WHO)

#### Policy stakeholders are uncertain about the sustainability of cMDA programs without additional external funding

In each country, policy stakeholders cited concerns about sustainability and sources of future funding as barriers to launching a new cMDA program. Several constructs contributed to the development of this theme, including *relative priority, available resources, financial cost,* and *a non-CFIR construct, donor relationships,* that captured concerns around the limited number of donors in the NTD space (Fig. 1)*.*

National-level policy stakeholders in Benin and Malawi were concerned that cMDA for STH would not be successful over the long-term without support from NGOs, donors, or other external organizations that choose to champion the potential policy change.

We may not have the resources to do mass drug administration so given a chance that there is an NGO which is trying to do the same, we always welcome the idea because it is complimenting government services and government is being supported. So, I have never seen a resistance concerning a programme like [cMDA] from the Ministry of Health. We do not do them because the resources are limited so we prioritize.—Malawi #3 (District)

In contrast, in India, resources were not perceived to be a major barrier, as national-level policymakers highlighted a sense of growing autonomy and reduced donor dependence (CFIR construct *available resources,* Fig. 1).

India is very well placed because it has its own resources, it is not donor dependent… There is a benefit to it also but there is also a slip side to it because it’s your own money…I know in countries like Nigeria, Ethiopia, obviously it’s hugely… donor dependent…the Governments do not have money of their own so the prioritization may also depend upon what priorities of donors are…and that does not necessarily have to be country driven always, we know that.—India #1 (Implementing partner)

Policymakers noted that the limited number of donors and partners supporting NTD programs globally might compromise the ability for programs to test and scale up innovative approaches to cMDA delivery. National-level policy stakeholders in India highlighted WHO and funders would need to continue to be engaged in research and implementation, following launch of a cMDA program.

WHO of course will continue to be an important player not just for drug donation but even for guiding…the guidelines are coming from WHO so how do we keep WHO informed about what India is doing…so that they can inform and guide us and then there is a donor community which is very small I think unfortunately for neglected tropical diseases at large…but there may be a few donors and…they might be the big ones but they are few in number. So how can the donor community be continued to be engaged on this so that without investments I don’t think any of this research building or trying new options can be possible.—India #1 (Implementing partner).

#### Concerns about existing intragovernmental partnerships are a barrier to launching cMDA

Transitioning to cMDA for STH will require varying degrees of system redesign in each country, presenting unique challenges in each site. The primary CFIR construct contributing to this theme is *cosmopolitanism*, highlighting opportunities and challenges for coordination between the MOH and other ministries, non-ministry partners, and other collaborators (Fig. 1).

In Malawi, partnerships between the MOH and other ministries may need to be formalized prior to launch of a cMDA program. This includes creating administrative structures to link ministries and designate lead ministries for launching cMDA. A challenge that several stakeholders at both national and sub-national levels in Malawi noted is a lack of formal information-sharing systems and supervisory structures to help the MOH effectively collaborate with other ministries, like the Ministry of Education (Fig. 1).

We need to start at the Ministry level. Where Ministry of Health and Ministry of Education and other Ministries that are relevant in implementation of this MDA. They need to integrate and to find a mechanism whereby there will be an integration in terms of calendars for example school, or administration of drugs. We need to have an understanding to say, at this time it's when we will be doing the MDA.—Malawi #9 (implementing partner)

In contrast, policy stakeholders in Benin and India did not mention cross-ministry or partner coordination as obstacles to the introduction of cMDA for STH. Stakeholders in both countries did, however, highlight the importance of launching cMDA as joint initiatives across ministries (Fig. 1).

Health is not the responsibility of the Ministry of Health alone; it is a multi-sectoral issue, and which requires the participation of almost all ministries. The treatment must not be done by the Ministry of Health alone. We must involve the Ministry of Education, Interior.—Benin #6 (Subnational)

#### Cross-cutting theme: future updates to STH policy will require rigorous evidence to ensure buy-in from policy stakeholders

Before launching a cMDA program, policy stakeholders indicated that rigorous clinical and implementation evidence are needed to inform any future updates to current STH policies. The primary CFIR constructs contributing to this theme were *knowledge and beliefs* about cMDA and *evidence strength and quality*, (Fig. 1).

Policy stakeholders in each country had strong positive attitudes towards launching cMDA driven by the belief that it could potentially eliminate STH transmission (CFIR construct *knowledge and beliefs*, Fig. 1).

It is a mass distribution to the whole population of the community. So, it will work because the drugs will be administered to all the targeted clients. So, the chances of the parasite surviving because sometimes it comes from one person to another, so if it will be distributed at the same time or given a period of time, I think it can be effective.—Malawi #9 (Implementing partner)

Stakeholders indicated that there is a dearth of evidence related to STH and transmission interruption. The evidence base will need to be strengthened before proposing new shifts in policy. Policy stakeholders prioritized clinical trial and cost-effectiveness data to inform potential updates to STH policy. Stakeholders in all countries also pressed that evidence about implementation and effective delivery of cMDA were necessary to launch cMDA (CFIR construct *evidence,* Fig. 1).

If you look at the publications on STH, they are not too many that you can find, and even if there are, they are small studies…continue to invest in the research I think will be important. – India #1 (Implementing partner)

The policies that we have currently they are school-based. And whatever information is in that, there are mostly school based and obviously it needs to change to cater for the community element. We would want to see if [cMDA] is successful or not. If it’s successful that’s where policies need to change because the research is there to guide policies makers.—Malawi #7 (District)

Policy stakeholders overall were encouraged by new cMDA clinical trials and were optimistic about incorporating evidence into future policies. Indian stakeholders were the most optimistic in terms of acknowledging potential pathways for scaling up a cMDA policy, while also acknowledging complexities with sustaining new programs (Fig. 1). Stakeholders from Benin believed that the standard of care for STH could be changed, but the availability of financial and human resources could act as a barrier to future policy changes (Fig. 1). Malawian stakeholders were more neutral in their responses about changing the standard of care, but notably many indicated enthusiasm for testing cMDA for transmission interruption (Fig. 1).

## Discussion

This study used formative qualitative research among policy stakeholders to identify the perceived barriers and facilitators to launching cMDA for STH transmission interruption in Benin, India, and Malawi (Table 2). Facilitators to the successful launch of cMDA were tailored community sensitization plans in place to proactively address local myths, improve intervention awareness, and achieve high treatment coverage. Policy stakeholders across all sites also noted that leveraging existing MDA infrastructure—including material and human resources—will be necessary for the effective launch of cMDA policies, so as not to duplicate existing programs. Barriers to the successful introduction of cMDA included overburdening the existing healthcare workforce to deliver cMDA, uncertainty regarding the sustainability of cMDA, and a need for greater collaboration across ministries and non-governmental partners before launch.

**Table 2:** Summary of key results by theme and country

Theme	Related CFIR constructs and other codes	Common findings across three sites	Country-specific findings		
			Benin	India	Malawi
*Key facilitators for launching cMDA*					
Effective, tailored community sensitization is a key component of successfully launching cMDA	Engaging participants Engaging leaders Design quality and packaging Intervention complexity	Strong outreach activities at baseline are particularly important for overcoming myths related to cMDA rollout	Outreach activities will need to be tailored to address adults who may believe they are at low risk for STH infection	Outreach activities will need to be tailored to address adults who may believe they are at low risk for STH infection Outreach activities should be further tailored by geography, education, and socio-economic status	Outreach messages need to be tailored to community sub-groups, such as religious groups
Opportunities to leverage existing health campaign infrastructure is important for policy stakeholders to support cMDA launch	Structural characteristics Tension for change Adaptability	Existing school-based and lymphatic filariasis MDA resources can be used to support the launch of cMDA including human resources and existing infrastructure	cMDA for STH should be integrated within child health programs	cMDA for STH should be integrated within maternal and reproductive health program objectives	No additional Malawi-specific findings
*Key barriers to launching cMDA*					
Policy stakeholders are concerned about health worker workload when implementing cMDA	Available resources Organizational incentives Implementation climate Relative advantage Intervention complexity Readiness	Stakeholders are concerned that Community Drug Distributors (CDDs) are overworked and that increasing workload will lead to poorly delivered cMDA programs	Stakeholders believe there are not enough CDDs available to support a cMDA program Insufficient incentives and a focus on performance-based targets are barriers to successfully launching cMDA Supervisors may not be able to provide adequate supervision to CDDs during cMDA programs	Stakeholders suggest that the CDD workforce would need to be increased to deliver cMDA and overcome challenges including delivery to hard-to-reach places The health workforce may not immediately accept launching cMDA if it increases their workload	Stakeholders believe there are not enough CDDs readily available to support a cMDA program Launching cMDA would require additional monetary incentives for the involved health workforce The CDD workforce would need to be expanded The cMDA program would need to account for challenges including delivery in hard-to-reach areas, inadequate time to deliver, and multiple visits
Policy stakeholders are uncertain about the sustainability of cMDA programs without additional external funding	Relative priority Available resources Financial cost Donor relationships	There are a limited number of donors and partners supporting NTD programs globally, which might compromise the ability to scale-up cMDA programs more broadly	cMDA will not be successful in the long-term without support from donors and non-governmental organizations	Financial resources were not perceived to be a major barrier due to reduced donor dependence	cMDA will not be successful in the long-term without support from donors and non-governmental organizations
Concerns about existing intragovernmental partnerships are barriers to launching cMDA	Cosmopolitanism	No common finding across all three countries	Did not highlight cross-ministry or partner coordination challenges	Did not highlight cross-ministry or partner coordination challenges	Stakeholders are concerned about the lack of formal information-sharing systems and supervisory structures necessary to collaborate between involved government ministries
*Cross-cutting theme*					
Future updates to STH policy will require rigorous evidence to ensure buy-in from policy stakeholders	Knowledge and beliefs Evidence strength and quality	Rigorous clinical and implementation evidence are needed before updating current STH policies Policymakers had positive attitudes that cMDA could potentially eliminate STH, primarily because they believed that tools are currently available to achieve targeted endpoints	No additional Benin-specific findings	No additional India-specific findings	No additional Malawi-specific findings

Stakeholders in all sites identified adaptive community sensitization as one of the most important determinants of successfully launching cMDA programs, due to its role in preparing communities. Without proper sensitization, cMDA could fail to achieve the coverage needed to stop STH transmission, potentially delegitimizing the program early in its implementation. The importance of community sensitization is well known in the MDA literature. For example, in the Greater Mekong Subregion, policymakers and scientists in a formative qualitative study on malaria elimination identified community engagement and sensitization as a priority for addressing the negative impact of rumors and misconceptions on future uptake of MDA [22]. A systematic review of community participation in the development and implementation of public health programs in high- and middle-income countries suggested that policymakers should consider investing resources in community sensitization and participation during program design, before implementation begins [23]. This mirrors findings from our study, emphasizing that strong community sensitization is not only a mid-implementation investment, but rather a key criterion for shaping policymaker decisions.

Opportunities to leverage existing MDA campaign infrastructure were perceived to be an asset for launching cMDA; economies of scope might be achieved if existing infrastructure could be utilized to expand deworming. However, policy stakeholders across sites noted that health workers were overstretched and that existing human resources may not be able to absorb a cMDA for STH program. A qualitative evaluation of cMDA for STH in Kenya found that community health volunteers and community members similarly identified health worker-workload and capacity as challenges to delivery [24]. Our study highlights that policy stakeholders may view cMDA as a feasible program due to existing infrastructure, but they are concerned that community-level implementers may not feel the same way. Incorporation of cMDA into existing health systems and devolution of tasks to CDDs will require careful planning to reach economies of scope without compromising the productivity of the health workforce in achieving cMDA objectives. Policy stakeholders in this study specifically highlighted that training and campaign infrastructure from LF programs could be leveraged to prepare CDDs to distribute albendazole. When determining the cost-effectiveness of cMDA, health economists should consider the additional costs needed to hire and train new health workers when expanding from school-based MDA to cMDA [25].

Launching successful cMDA for STH is reliant upon leveraging external funding that currently supports school-based deworming programs. One concern amongst stakeholders in Benin was the cost of drugs needed for cMDA in the future, should drug donations ultimately be halted. NTD policy stakeholders have observed this challenge in other settings; for example, onchocerciasis programs targeting disease elimination in Africa have often had setbacks due to inadequate domestic funding. Programs continue to need partnerships and financial support to achieve elimination [26]. Cost and resource barriers may lead policy stakeholders to believe that the program will not be sustained within their local context. If policy stakeholders do not believe that a program can be sustained in the long term, they may be less likely to support the launch of cMDA in the short term.

Policy stakeholders in Malawi described a lack of intra-governmental coordination and collaboration in standard of care school-based MDA. In contrast, stakeholders in Benin and India highlighted opportunities for joint intra-government initiatives to support launch of cMDA. A study of Bihar’s school-based deworming program highlighted the importance of collaboration between health and education departments: where infrastructure was shared, the program achieved higher coverage and reduced overall program costs [27].

Policy stakeholders also stressed that any future policy changes need to be grounded in rigorous trial evidence about STH transmission and implementation evidence such as cost-effectiveness, not simply driven by political momentum. For example, a triple drug regimen for LF moved rapidly from evidence to policy due in part to the availability of both clinical trial evidence and acceptability studies [28, 29]. Further, a study on national policy development found that evidence translation was expedited when research included operationalization and implementation questions, which was also echoed by policy stakeholders in this study [30]. When considering launch of cMDA it is necessary to provide policy stakeholders with both clinical and implementation evidence to move from evidence to launching of new evidence-based programs.

There are several strengths of this study. This study included a sample of policy stakeholders from three countries before implementing a multi-site randomized clinical trial. This enabled our study team to engage with policy stakeholders and understand their perspectives on cMDA prior to study launch. Our study team used the CFIR from end-to-end, from data collection to analysis, and previous systematic reviews have demonstrated a dearth of studies using the CFIR in such a comprehensive manner [31]. Further, study coders were able to determine if each CFIR construct was a facilitator or barrier for each stakeholder, leading to the identification of constructs most influential in influencing acceptability of launching cMDA.

This study also has several limitations. Policy stakeholders knew that cMDA would soon be implemented in certain geographic areas by the DeWorm3 Project and, as a result, their responses may have been affected by social desirability bias. Further, policy stakeholders may have been hesitant to disclose major challenges of launching cMDA, due to concerns related to future funding or external support. To preemptively address these concerns, policy stakeholders were told prior to interviews that their responses would not impact policy decisions or funding. Also, because each site tailored the individual interview guides to account for local implementation contexts and cultural differences, there may be differences in how questions were asked across sites. Lastly, several transcripts were not coded using double coding, and biases related to the interpretation of the data are possible.

## Conclusions

This formative study applied the CFIR to describe perceived barriers and facilitators of launching cMDA for STH transmission interruption, from the perspective of STH policy stakeholders in Benin, India, and Malawi. Policy stakeholders noted several challenges, primarily human and financial resource constraints that need to be accounted for should future STH for cMDA be launched in routine NTD programs. However, findings indicate that cMDA for STH was broadly appealing to policy stakeholders because it can leverage routine health program resources and contribute to improved maternal and child health. These findings indicate that policy stakeholders largely support launching cMDA for STH and are cautiously optimistic that it might effectively interrupt STH transmission. Continued communication with policy stakeholders will provide the opportunity to understand how perceptions of cMDA for STH may change as stakeholders are engaged in implementation.

## Supplementary Information

**Additional file 1: Appendix 1.** COREQ Table

## Data Availability

The data that support the findings of this study are available from the corresponding author, ARM, upon reasonable request.
