# Supplementary material for: Policy stakeholder perspectives on barriers and facilitators to launching a community-wide mass drug administration program for soil-transmitted helminths
Source: Glob Health Res Policy. 2022 Dec 2;7:47. doi: 10.1186/s41256-022-00281-z (PMC9716752; doi:10.1186/s41256-022-00281-z)
Supplement: Supplementary file 1 — Additional file 1: Appendix 1. COREQ Table [file 41256_2022_281_MOESM1_ESM.docx]

**Appendix 1.** COREQ Table

| **No** | **Item** | **Guide questions/description** | **Response/Page number referenced** |
| --- | --- | --- | --- |
| **Domain 1: Research team and reflexivity** | | | |
| Personal Characteristics | | | |
| 1. | Interviewer/  facilitator | Which author/s conducted the interview or focus group? | Page 18 Author contributions |
| 2. | Credentials | What were the researcher's credentials? E.g., PhD, MD | Page 1 Author list  AR (MPH), MS (MSc), EO (BA), AT (MSW), SKJ (PhD), MCGC (MSc, MHS), EA (MD), FC (MSc), CIT (MSc), ABE (PhD), PN (MSc), JLW (MD), SSRA (MD, PhD), MI (PhD), KK (MD), KA (MD), ARM (PhD) |
| 3. | Occupation | What was their occupation at the time of the study? | Page 1 Author list  AR (Implementation Science (IS) Data Analyst), MS (DeWorm3 Social Scientist), EO (IS Data Analyst), AT (DeWorm3 Social Scientist), SKJ (Professor and Head, VRHP, KEM), MCGC (Research Assistant, PhD Candidate), EA (DeWorm3 site deputy coordinator), FC and CIT (DeWorm3 IS Assistants), ABE (Research Assistant), PN (DeWorm3 IS Assistant), JLW (DeWorm3 study PI; Professor University of Washington), SSRA (Professor, The Wellcome Trust Research Laboratory, Division of GI Sciences, CMC, Vellore), MI (DeWorm3 site PI; Professor of Parasitology at University of Abomey-Calavi), KK (DeWorm3 site PI); KA (DeWorm3 Implementation Science Coordinator); ARM (DeWorm3 Implementation Science PI, Assistant Professor University of Washington) |
| 4. | Gender | Was the researcher male or female? | Both male and female researchers were involved. However, this is not relevant in the context of this study as would not have affected the conduct or analysis of individual interviews on facilitators and barriers to launching a cMDA for STH program. |
| 5. | Experience and training | What experience or training did the researcher have? | Page 2 (Methods section): “All coders were trained on data analysis using standard operating procedures and standardized analysis plans.” |
| Relationship with Participants | | | |
| 6. | Relationship established | Was a relationship established prior to study commencement? | Participants were selected from DeWorm3 stakeholder lists. No prior relationship established with participants prior to study commencement. |
| 7. | Participant knowledge of the interviewer | What did the participants know about the researcher? e.g., personal goals, reasons for doing the research | Participants knew the purpose of the DeWorm3 study. |
| 8. | Interviewer characteristics | What characteristics were reported about the interviewer/facilitator? e.g., Bias, assumptions, reasons, and interests in the research topic | The individual interviews were conducted by experienced qualitative researchers specifically trained on DeWorm3 question guides and interview process. |
| **Domain 2: study design** | | | |
| Theoretical framework | | | |
| 9. | Methodological orientation and Theory | What methodological orientation was stated to underpin the study? e.g., grounded theory, discourse analysis, ethnography, phenomenology, content analysis | Page 2 (Methods section): “This analysis is informed by the Consolidated Framework for Implementation Research (CFIR), a meta-theoretical framework of 38 constructs that provides a typology of constructs for characterizing potential determinants (both barriers and facilitators) to implementation from the perspective of individuals involved in implementation.”  Page 3 (Methods section): “Case memos were developed for each stakeholder group in each site. Memos included a summary of how each code was applied, code patterns, and demonstrative quotes…Heat maps were used to visualize coded qualitative data, and delineate perceived facilitators and barriers, by interviewee and CFIR construct. Where the individual indicated that the construct would make the introduction of cMDA easier, their response was coded as a strong (dark green) or moderate (light green) facilitator. Where the individual indicated that the construct would make the introduction of cMDA difficult, their response was coded as a strong (dark blue) or moderate (light blue) facilitator. If the individual gave contradictory remarks about a construct, it was coded as mixed (dark orange). Constructs had a neutral influence (light orange) on cMDA if it appeared to be purely descriptive or if there was no evidence of positive or negative influence. Facilitators and barriers were identified by noting patterns that were cross-cutting across individuals, constructs and/or countries.” |
| Participant selection | | | |
| 10. | Sampling | How were participants selected? e.g., purposive, convenience, consecutive, snowball | Page 2 (Methods section): “Stakeholder mapping workshops were conducted to identify people who were considered critical change agents at the national, state, and district levels. Purposive quota sampling was used to select interviewees in each country across stakeholder “levels”, including WHO country offices and implementing partners, national government personnel, and sub-national government personnel (Table 1).” |
| 11. | Method of approach | How were participants approached? e.g., face-to-face, telephone, mail, email | Page 2 (Methods section): In-person individual interviews.  “Interviews were conducted in private locations with a facilitator and notetaker present.” |
| 12. | Sample size | How many participants were in the study? | Page 2 (Methods section): The number of study participants is outlined in Table 1: Stakeholders by country and level (n=40). |
| 13. | Non-participant | How many people refused to participate or dropped out? Reasons? | No participants refused. |
| Setting | | | |
| 14. | Setting of data collection | Where was the data collected? e.g., home, clinic, workplace | Page 2 (Methods section): “Interviews were conducted in private locations with a facilitator and notetaker present.” |
| 15. | Presence of non-participants | Was anyone else present besides the participants and researchers? | Not applicable, no other groups were present during conduct of focus group discussions. |
| 16 | Description of sample | What are the important characteristics of the sample? e.g., demographic data, date | Page 2 (Methods section): “Stakeholder mapping identified people who were considered critical change agents at the national, state and district levels.” |
| Data collection | | | |
| 17. | Interview guide | Were questions, prompts, guides provided by the authors? Was it pilot tested? | Page 2 (Methods section): “The CFIR informed the design of six semi-structured interview guides with a mix of respondent and informant style questions, tailored to each stakeholder group across partner, national, state, district and sub-district levels.” |
| 18. | Repeat interviews | Were repeat interviews carried out? If yes, how many? | Not appliable – repeat interviews were not conducted. |
| 19. | Audio/visual recording | Did the research use audio or visual recording to collect the data? | Page 2 (Methods section): “Following consenting and assenting procedures, all FGDs were audio-recorded.” |
| 20. | Field notes | Were field notes made during and/or after the interview or focus group? | Page 2 (Methods section): “Interviews were conducted in private locations with a facilitator and notetaker present.” |
| 21. | Duration | What was the duration of the interviews or focus group? | Duration of individual interviews were between 35 and 80 minutes. |
| 22. | Data saturation | Was data saturation discussed? | Determination of data saturation was not an objective of this research study. Data saturation was reached within sites; however, heterogeneity was present across sites. |
| 23. | Transcripts returned | Were transcripts returned to participants for comment and/or correction? | Transcripts were not returned to study participants. |
| **Domain 3: analysis and findings** | | | |
| Data analysis | | | |
| 24. | Number of data coders | How many data coders coded the data? | Page 2 (Methods section): “A group of 5 coders based in Seattle, Washington, United States, Vellore, India and Cotonou, Benin engaged in data analysis.” |
| 25. | Description of the coding tree | Did authors provide a description of the coding tree? | A coding tree was not made for this study. |
| 26. | Derivation of themes | Were themes identified in advance or derived from the data? | Page 2 (Methods section): “The codebook was prepared a priori, based upon the CFIR constructs, and a mix of deductive and inductive coding was used. The codebook was updated iteratively by the coders to provide inclusion of inductive themes, including donor relationships, gender, and equity, during the coding process.” |
| 27. | Software | What software, if applicable, was used to manage the data? | Page 2 (Methods section): “Atlas.ti 8 qualitative software was used to store and organize the transcripts.” |
| 28. | Participant checking | Did participants provide feedback on the findings? | Participants did not provide feedback on the findings. |
| Reporting | | | |
| 29. | Quotations presented | Were participant quotations presented to illustrate the themes / findings? Was each quotation identified? e.g., participant number | Page 3-9 (Results section): Quotations are presented throughout the text alongside interpretations. |
| 30. | Data and findings consistent | Was there consistency between the data presented and the findings? | Page 3-9 (Results section) and Page 10-12 (Discussion section): Quotations are presented throughout the text in the Results section alongside interpretations. The meaning of quotations was further explored in the Discussion section. |
| 31. | Clarity of major themes | Were major themes clearly presented in the findings? | Page 3-9 (Results section): Major themes are presented and organized as key facilitators, barriers, and cross-cutting themes that affect the implementation of cMDA. |
| 32. | Clarity of minor themes | Is there a description of diverse cases or discussion of minor themes? | Page 3-9 (Results section): Minor themes are discussed in more detail through quotations and interpretations under major theme headings. |
